# Laser propulsion of nanobullets by adiabatic compression of surface plasmon polaritons

**DOI:** 10.1038/srep17652

**Published:** 2015-12-03

**Authors:** Viola Folli, Giancarlo Ruocco, Claudio Conti

**Affiliations:** 1 Department of Physics, University Sapienza, Piazzale A. Moro, 5, 00185, Rome, Italy; 2 IPCF-CNR, UOS Roma Sapienza, Piazzale Aldo Moro 5, 00185, Rome, Italy; 3 Fondazione Istituto Italiano di Tecnologia (IIT), Center for Life Nano Science Viale Regina Elena 291, 00161 Rome, Italy; 4 ISC-CNR, UOS Roma Sapienza, Piazzale Aldo Moro 5, 00185, Rome, Italy

## Abstract

Laser propulsion and guide of nanosized objects is fundamental for a wide number of applications. These applications are often limited by the fact that the optical forces acting on nanoparticles are almost negligible even in the favorable case of metallic particles and hence large laser powers are needed to accelerate and guide nanosize devices in practical applications. Furthermore, metallic nanoparticles exhibit strong absorption bands and scattering and this makes more difficult controlling nanopropulsion. Thus, finding some mechanism enhancing the optomechanical interaction at the nanoscale controlled by laser is specifically challenging and pivotal. Here, we demonstrate a novel physical effect where the well-known adiabatic localization of the enhanced plasmonic surface field on the apex of metallic nanocones produces a significant optical pressure employable as a propulsive mechanism. The proposed method gives the possibility to develop new photonics devices to accelerate metallic nanobullets over long distances for a variety of applications.

In the last decades, the ability to propel and precisely guide metallic particles with nanoscopic dimensions^1^ has found an increasing attention in all the field of nanosciences, for the broad spectrum of interdisciplinary applications and related challenges ranging from nanobiotechnology and nanomedicine to nanoelectronics and communications^2^,^3^,^4^. There have been so many developments on this topic over the past years regarding the plasmonics enhanced optical forces, showing how the necessity to precisely guide nanoparticles represents one of the main topic of nanoscience^5^,^6^,^7^,^8^. Nanoplasmonics involves a wide range of possible promising scientific applications in the branch of optical manipulation. In fact, conventional optical manipulation is limited by the light diffraction limit that prevents the confinement of the light beyond wavelength fraction, causing the trap to be unstable. Evanescent fields can be focused beyond the diffraction limit and have been recently employed to optical trapping of nanometric structures. Among several types of evanescent waves, the use of surface plasmons, that are enhanced evanescent waves, allows to largely intensify the optical forces experienced by the trapped particles, opening the road to control and manipulation of nanometre-sized objects in a particularly efficient way. In fact, the plasmonic nature of metal nanoparticles allows to employ the surface plasmons polaritons to further increase optical force fields and to create a stable trap much more stronger (about 40 times stronger than normal evanescent waves) and at much lower power (about three orders of magnitude)^9^,^10^,^11^,^12^. Specifically, the optical manipulation of gold nanoparticles is widely used and particularly useful in biophysics and medicine due to their unique properties charming both in spectroscopy and in chemistry. Gold nanoparticles show strong resonant optical properties due to the formation of surface plasmon polaritons (SPPs) that highly enhance the Raman scattering and hence are largely used in several forms of enhanced Raman spectroscopy (ERS)^13^,^14^,^15^. The enhancement factors of Raman scattering signal can be found in the range of 10^10^–10^11^ and are strong enough to allow single molecule ERS detection^16^,^17^,^18^. Jointly, from a chemical point of view, gold is one of the most used materials for bioscience interfaces. In fact, gold nanoparticles are easily funcionalized and introduced deeply in the human body where can be used for phototermal therapy, smart drug delivery and several non-invasive cancer treatments^19^,^20^. Further, gold nanoparticles can be strongly controlled over shape and size giving a wide assortment of their chemical, optical and electromagnetic properties^21^. The rich variety of phenomena related to metallic nanoparticles (like plasmonic resonances, local heating, fluorescence enhancement) gives a complex framework for light behavior. However, critical issues are related to the manipulation and controlled propulsion of nanoscopic metallic particles. As for example, a big challenge in biosciences and nano molecular medicine is the punctual delivery of nanoparticles inside the living cells reducing the cell death rate related with high laser intensities^22^. Using the enhanced optical force of the resonant plasmonic field allow to considerably reduce the laser input peak power and to employ the strong field enhancement due to plasmon resonances for membrane photoporation and autopropulsion of gold nanoparticles inside the cell. This can represent a big improvement in the optical manipulation and nanodelivery.

In the following, we first discuss about the kinetic momentum associated to a tapered plasmonic waveguide which design displays an effective refractive index with singularity on the tip. Next, we numerically simulate the plasmonic field evolution on the metallic cone, observing that a sign change happens in the optical pressure when nanofocusing is enabled by the waveguide geometry. We provide numerical demonstration that the adiabatic concentration of surface plasmonic polaritons at the nanoscale is the leading mechanism inducing an overall positive optomechanical force. Finally, we furnish the fully analytical description of the role of adiabatic compression of surface plasmon polaritons in the optical force through the Maxwell stress tensor method and verify the applicability of this novel effect in practical issues.

## Results

### The kinetic momentum of adiabatic compression

**Figure 1:**
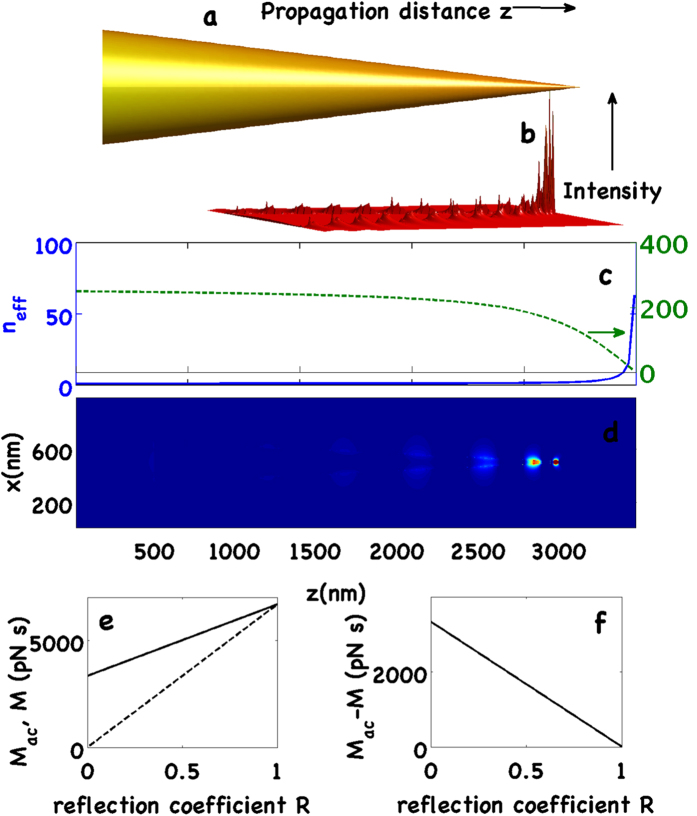
(**a**) Sketch of a conical waveguide sustaining the SPP adiabatic compression. The propagation direction *z* is indicated by the arrow. (**b**) FDTD simulations: plasmonic field intensity in the *xz* plane. (**c**) Analytical solution of equation (1) for the SPP effective index (left axis) and phase velocity (right axis) as a function of the propagation distance *z*. (**d**) Snapshot of the normal component *E*_*x*_ of the electric field in the *xz* plane. (**e**) Transferred momentum versus the reflection coefficient 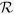, in the absence *M* (dashed line) and in the presence *M*_*ac*_ (continuous line) of adiabatic compression. (f) The difference *M*_*ac*_ − *M* versus 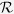.

One of the most important effects in nano-optics is a specific transport mechanism of electromagnetic (EM) energy at the nanoscale investigated in two seminal works^23^,^24^ and known as adiabatic compression (AC) of surface plasmons polaritons (SPPs)^25^,^26^,^27^,^28^. This process induces a huge energy concentration at the apex of metallic nanostructures, like, specifically, conical waveguides, and consists in an adiabatic progressive accumulation of the SPP field during the propagation towards the cone tip. This may be described in terms of an effective refractive index *n*_*eff*_ increasing along the waveguide axis; correspondingly, the SPP phase and group velocities tend to zero when approaching the apex. A strong localization of the optical field occurs without loss of energy [Fig. 1, panels (a)–(d)]. According to the following arguments, the SPP enhancement suggests a possible involvement of AC in the optical pressure acting on the nanostructure. In the case of a dielectric box, the “Balazs Block”^29^,^30^,^31^,^32^, any photon transmitted through the device produces a displacement of the block and does not trasfer momentum to the matter after the interaction because of the energy-momentum conservation; indeed, the momentum of the transmitted photon at the output is equal to the momentum of the photon at the input. If AC is present, ideally, the photons do not exit the device, and get localized in the SPP at the cone tip; as a result it may be expected that their initial momentum is transferred to the metallic cone. Correspondingly, the onset of the AC may enhance the optically induced force on a plasmonic waveguide. In addition, any reflected photon also contributes to the mechanical momentum of the object. And in the case of AC, reflection also occurs at the surfaces of the cone, further increasing the optomechanical action. This effects are analyzed in this Letter by finite-difference time-domain (FDTD) simulations, and by calculating the Maxwell stress tensor (MST). We consider again the Balazs block: a photon with energy *ħω*, has a reduced velocity *c*/*n* when entering a dielectric block with refractive index *n*, and propagation length *L*. No mechanical forces act on the material box during the transit time Δ*t* = *nL*/*c*. At the photon entrance a force pushes forward the block, and, when leaving the box, the photon generates a recoil force (see, e.g., the simulations in^32^).

In a metallic conical waveguide [Fig. 1, panel (a)], letting *z* the cone axis and propagation distance, the effective refractive index *n*_*eff*_ (*z*) satisfies the equation^23^


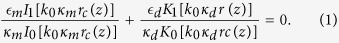


with *r*_*c*_(*z*) the local cone radius, *I*_*p*_ and *K*_*p*_ modified Bessel functions (*p* = 0, 1), *k*_0_ = 2*π*/*λ*, 
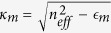
, 
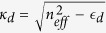
, 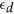 the dielectric constant of the medium surrounding the cone, and 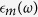 the Drude-Lorentz dielectric function of the metal. For cone made of gold in air, with base *R* = 300 nm, height *h* = 2500 nm, and wavelength *λ* = 532 nm, as shown in Fig. 1c, when approaching the apex, the real part of the analytical solution of equation (1) largely increases (continuous line) and the phase velocity (dashed line) tends to zero. The transit time 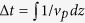 tends to infinity and the SPP is adiabatically compressed. In Fig. 1b, we show a snapshot of the electric field intensity in the *xz* plane. The intensity on the tip is enhanced by more than three order of magnitudes with respect to value at the cone base. In Fig. 1d, we show the corresponding *E*_*z*_ component, revealing the compression towards the tip. When considering the optical forces, we first observe that the kinetic momentum^31^ transferred by a reflected photon to the block is 2*ħω*/*c*, and the transmitted photons do not furnish kinetic momentum. Considering 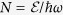 photons, being 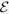 the total energy of the light beam, and assuming a reflection coefficient 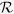, the momentum transferred by the 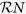 reflected photons is 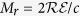. In the presence of AC, the transmitted fraction of photons 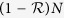 is trapped on the tip (ideally, the time needed to pass through the cone is infinite). The total momentum transferred to the block from the compressed photons is 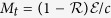. From these arguments, in the presence of AC, the total momentum gained by the block is 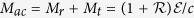. Being 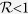, this quantity is always greater than in the case of dielectric block for which 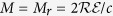 [see Fig. 1 panels (e,f)]. AC enhances the mechanical momentum transferred to the block. However, these arguments are extremely simplified. In the following, we resort to first principles solutions of the Maxwell equations.

### Numerical simulations

**Figure 2:**
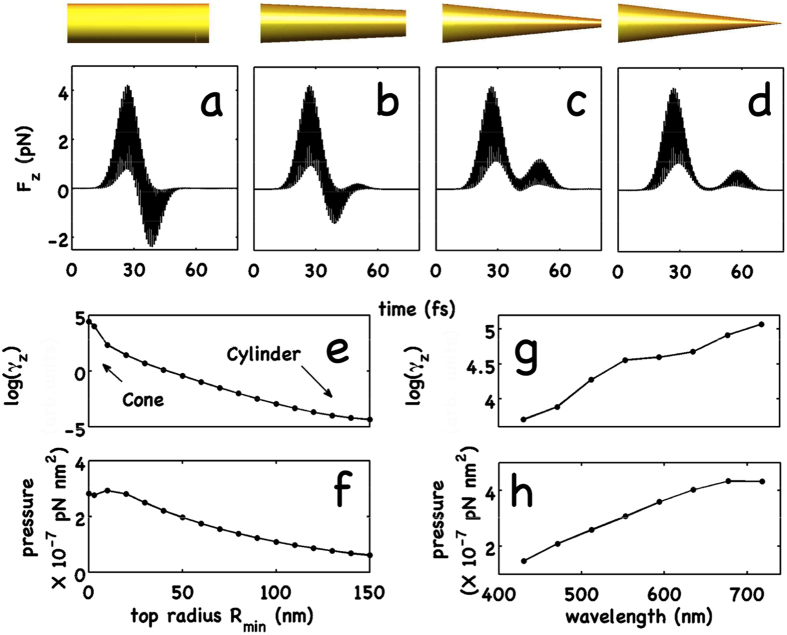
(**a**–**d**) Calculated force *F*_*z*_(*t*) versus time in the presence of a pulsed radially polarized excitation (pulse duration *T* = 10 fs, peak power *P* = 1 mW) for a cylinder, for frustum cones with *R*_*min*_ = 100,30 nm, and for a cone (*R*_*min*_ = 0), in panels a,b,c, and d, respectively. The device geometries are sketched above the corresponding panels. (**e**) Logarithm of the enhancement *γ*_*z*_ of *E*_*z*_-component versus the top radius *R*_*min*_; (**f**) optical pressure versus the top radius *R*_*min*_. (**g**) Logarithm of *γ*_*z*_ versus the wavelength *λ* of the input pulse for cone; (**h**) optical pressure versus *λ*.

The investigation on the mechanical properties of AC has been carried out by a parallel FDTD algorithm^33^. We solve the full Maxwell equations in three spatial dimensions and time, including material dispersion by a Drude-Lorentz model for the metallic dielectric function, 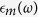, and adopting perfectly matched layer boundary conditions. The metallic waveguide is centered in the computational box with the cone axis placed in the *z*-axis. The nanocone has base radius *R* = 150 nm and height *h* = 2500 nm. These dimensions give an apex angle of 0.12 radiants and allow AC of SPPs^34^. The base of the cone is irradiated by a radially polarized Gaussian laser beam propagating along the cone axis, with wavelength *λ* = 532 nm and waist *w*_0_ = 400 nm. The opto-mechanical force is calculated through the flux of the MST across an ideal box strictly containing the nanocone. In order to give evidence of the connection between AC and optical pressure, we have performed simulations by varying the geometrical characteristics of the waveguide. Specifically, we have started from a cylinder and then we considered a frustum cone with decreasing apex radius *R*_*min*_ with respect to the base radius *R*. This is sketched in Fig. 2, notice that in the case of cylinder (*R*_*min*_ = *R*) AC does not occur.

In Fig. 2, we show the time dynamics of the longitudinal component of the instantaneous force, *F*_*z*_(*t*), for a pulsed excitation (similar results are obtained for the CW waves, not reported). Fig. 2, panels (a,d), shows *F*_*z*_(*t*) for a cylinder with *R* = 150 nm, and for frustum cones with top radius *R*_*min*_ = 100 nm, *R*_*min*_ = 30 nm, and *R*_*min*_ = 0. All the waveguides have heigth *h* = 2500 nm. The first peak in Fig. 2a corresponds to the entrance of the pulse at the base of the waveguide, the second peak is related to the exit of the pulse from the structure. Figure 2e shows the logarithm of the field enhancement *γ*_*z*_ of *E*_*z*_-component of the electric field, defined as the ratio between *E*_*z*_ at the waveguide tip and its value at the base. Figure 2f shows the optical pressure as a function of the apex radius *R*_*min*_, from a cone (*R*_*min*_ = 0) to a cylinder (*R*_*min*_ = *R*). The optical pressure is defined as the time-averaged force per unit of transverse surface in the *z*–direction. In a cylinder, as for the Balazs block^32^, the second peak of the force has an opposite sign with respect to the first one corresponding to the recoil force described above (see Fig. 2a,b). In the presence of AC (Fig. 2c,d), the recoil is absent. The nanostructure is subject to a second force in the same direction of the EM field. As a result, AC has an enhanced propulsive effect on the nanocone and on the total optical pressure (Fig. 2f). We also observe that, in the presence of AC, the second peak is retarded with respect to the cylinder because of the slowing down of the SPP.

We also performed numerical simulations by changing the wavelength of the input pulse. As it is shown in Fig. 2, panels g, h, the optical pressure and the electric field enhancement are strictly related. An increasing in AC efficiency with *λ* induces a corresponding enhancement of the optical pressure.

### Maxwell stress tensor for SPP

For electromagnetic waves with **E** and **H**, the expression for the time-dependent force is^35^


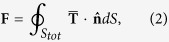


where 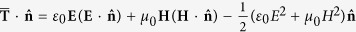 is the Maxwell Stress Tensor (MST), 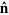 is the normal versor pointing outwards from the surface *S*_*tot*_ that encloses the nanostructure. From this expression, we have to calculate the time average over an optical cycle and the real part of the integration. Applying Eq. (4) to a *TM*_0_ mode (fully compatible with conical geometry and maximally efficient for adiabatic compression) the total optical force along the *z*-axis and acting on the gold nanobullet due to the propagation of the SPPs on its surface is given by


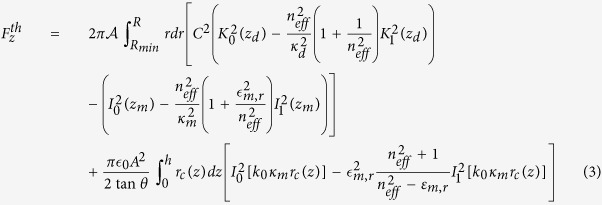


with 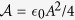, *z*_*m*_ = *k*_0_*κ*_*m*_*r* and *C* = *I*_0_(*k*_0_*κ*_*m*_*R*)/*K*_0_(*k*_0_*κ*_*d*_*R*), *z*_*d*_ = *k*_0_*κ*_*d*_*r*, where 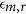 is the relative permittivity of the cone and 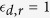. For what concerns the lateral contribution, we have defined the angle *θ* as 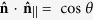 and 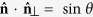 and we have integrate the flux of the MST through the element of surface *d*Σ = *r*_*c*_(*z*)*dφdz*/sin*θ* (see Methods sections for details). The relation between the angle *θ* and the top and base radii is *θ* = *π*/2 − arctan[(*R* − *R*_*min*_)/*h*] while *r*_*c*_(*z*) = [(*h* − *z*)/*h*]*R* represents the local radius of the nanobullet with height dependence. For more details concerning the analytical treatment of the optical force, we refer the reader to the Methods section.

**Figure 3:**
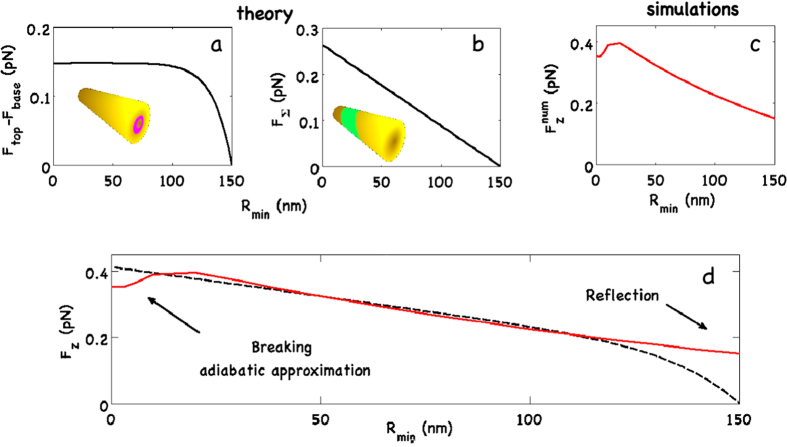
Theoretically calculated *F*_*z*_ from the entrance and exit faces (**a**) and from the lateral surface *S*_∑_ (**b**) versus the top radius *R*_*min*_. (**c**) Numerically calculated overall 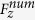 as a function of top radius. (**d**) Comparison of theoretical 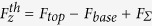 (dashed line) and simulations (continuos red line).

In Eq. (2), we distinguish two contribution, the first integral represents the optical force acting on the input and exit faces (*F*_*top*_ and *F*_*base*_), the second integral indeed is related to the lateral surface (*F*_Σ_). When decreasing the top radius *R*_*min*_, *F*_*top*_(*R*_*min*_) → 0 while the lateral contribution *F*_Σ_(*R*_*min*_) increases. Vice versa, approaching to the cylindrical geometry *θ* → *π*/2, for symmetry *F*_Σ_(*R*_*min*_) → 0 while *F*_*top*_ → −*F*_*base*_. In Fig. 3, we report the comparison between FDTD simulations and theory. Discrepancies appear in the limit of an ideal cone and for a cylinder. In the latter case, simulations and theory differ because of the FDTD calculation of the overall force, at difference with theoretical analysis, takes into account reflection and the force on the exit face is smaller than that on the entrance face. In the former case, the discrepancy is related to the limits of validity of adiabatic approximation. We remark that, when considering a cone, the recoil force at the top vanishes, but also an additional positive longitudinal component of the force appears due to adiabatic compression of surface plasmon polaritons on the lateral surface; this explains the additional positive force pulse in Fig. 2c,d with respect to Fig. 2a,b. With an input power coupled to the cone of about ~1 mW, we obtain an overall positive force pushing forward the cone of the order of *pN*.

We stress that the enhanced optical pressure acting on the whole conical structure is a phenomenon strictly related (and due) to the nano focused localization of the SPPs, and it disappears completely when the conditions for AC are not longer satisfied. We repeat the same analysis (not reported) for dielectric strongly absorbing structures to exclude other mechanisms. The enhancement effect of the averaged optical force disappears. We conclude that the observed enhancement of the propulsive optical force is strictly dependent from adiabatic compression of the plasmonic field. Furthermore, being the adiabatic compression of surface plasmon polaritons due to the real part of refractive index, the optomechanical effect of the enhanced propulsion in nano cones is a mechanism completely unrelated to absorption.

## Discussion

In conclusion, we have reported on the direct analysis of the opto-mechanical forces acting on nanosized metallic conical tip in the presence of AC of SPPs. We found that the presence of AC affects the overall optical pressure, increasing the total force acting on the tip surface. We have demonstrate and show a novel mechanism of auto-propulsion of nanosized object that can found wide-ranging applications in several fields of nanotechnology and nano-optics. This phenomenon is resulting from an interplay between the optical forces and the surface plasmons polaritons adiabatic compression.

**Figure 4:**
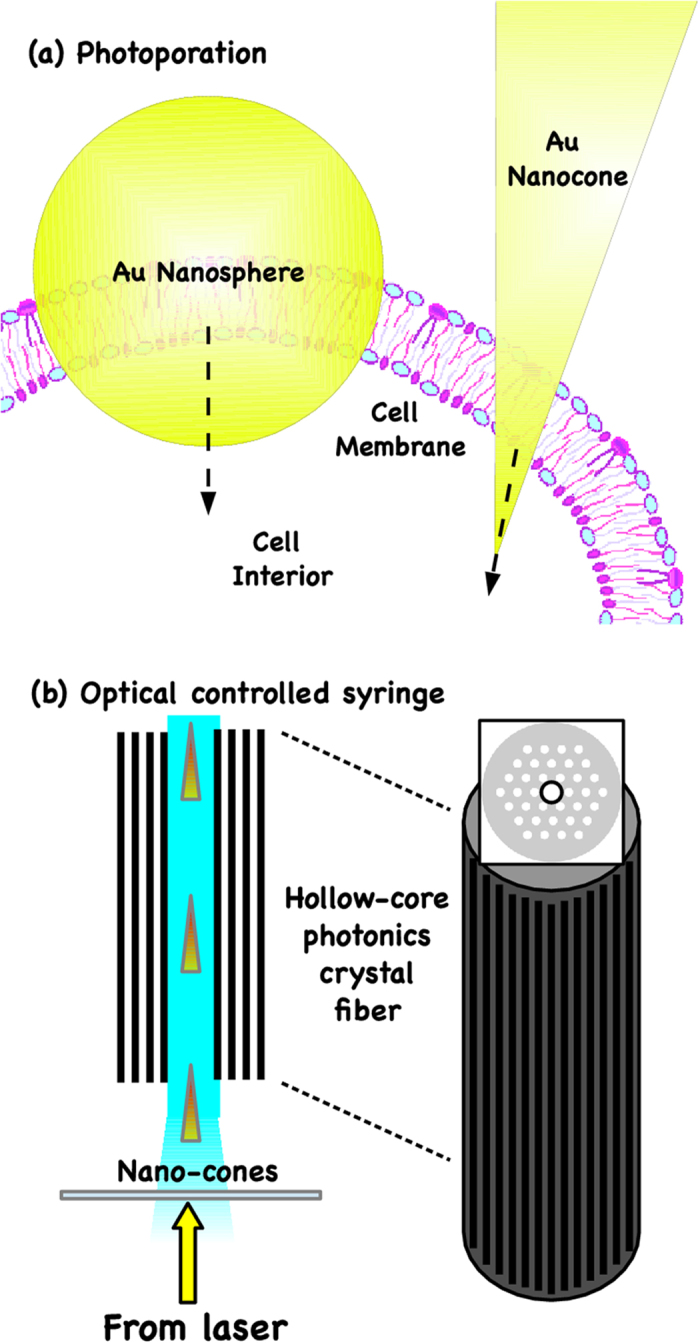
Examples of applications of light-propelled nanocones. (**a**) Photoporation of cell membrane (5–10 nm), (**b**) Optically controlled syringe of nanobullets.

Our study is relevant for the possible use of AC as a propulsive mechanism of nanosized objects. Applications may be envisaged, for example, in the field of gene or drug-delivery where the optically accelerated nanocone could be used for localized treatments, and in the control of the motion of optically activated nanodevices. For example, in applicative terms, the hotspot formation in conical or quasi-conical geometries due to adiabatic compression can be used for the efficient generation of cell membrane pores in laser-assisted photoporation [see Fig. 4, panel (a)]. In fact, we expect that the strong heating, nearly localized around the cone apex, can be used for the mechanical disruption of the cell membrane and the subsequent cone-injection directly into the living cell. In an attractive work^22^, Li *et al.* shows the controlled injection of 80 nm gold nanospheres into a living mammalian cell by combining the optical forces and plasmonic heating and reducing the cell death rate thanks to the reduction of laser power. The usage of adiabatic compression, no present in spherical geometry, can further increase the local heating together with a remarkable lowering of the pump power of driving laser. In fact, in^22^, they work with a minimum laser power of 5 mW, need to obtain a surface temperature of gold particle above the spinodal decomposition temperature of water (~320 °C). This induces the formation of a vapor-shell around the sphere that jointly protect and locally pierces the cell membrane. Using a gold cone gives higher local heating already at 1 mW. The adiabatic compression and the strong field enhancement on the tip (three or more order of magnitude) should induce the formation of nanoshells of vapor at lower input laser power and this results in a further increased cell viability. Furthermore, the cone geometry allows to reduce the puncture surface for photoporation from tens of nanometers (in a sphere) to some nanometers (apex dimension in the cone) but not the overall surface (worthwhile for chemical and biological functionalization). Finally, the positive optical force (pN at just 1 mW) can be used to propel and transfixes the nanocone on the cell membrane (5–10 nm thickness), reducing the laser exposure time.

Another potential application of AC-induced pressure is the optical guidance and propulsion of nanobullets inserted in hollow-core photonics crystal fibers (HC-PCF)^37^ to smart drug delivery [see Fig. 4, panel (b)]. In fact, the realization of a device able to inject metallic nanocones, drug-filled, into a single specific cell, sited in deepness, can represent a frontier for the smart-drug delivering where specific and local drug treatment are requested. At date, the smart-drug delivery with magnetic nanoparticles is possible by using magnetic fields, focused on the targeted sites, that capture the particles and extravasate them at the target. This method is effective only for targets close to the body’s surface, as the magnetic field strength falls off rapidly with distance. The realization of optically controlled syringes allows to inject directly in the target-sites the nanoparticles, also if they are placed in deepness. To this kind of device, the use of AC to guide metallic nanocones is fundamental because it allows to overcome the difficulties to trap and move nano-objects.

Another possible employment of our results is more specifically academic. At date, it does not yet exist a way to measure adiabatic compression (AC) and confirm the related Stockman’s theory. The optical propulsion, present only in conical adiabatically-compressive geometry, could represent the first experimental evidence of adiabatic compression, and the measure of the total force magnitude as a function of the apex radius could give the signature of the predicted AC regime.

## Methods

### Theoretical calculations of the optical forces

**Figure 5:**
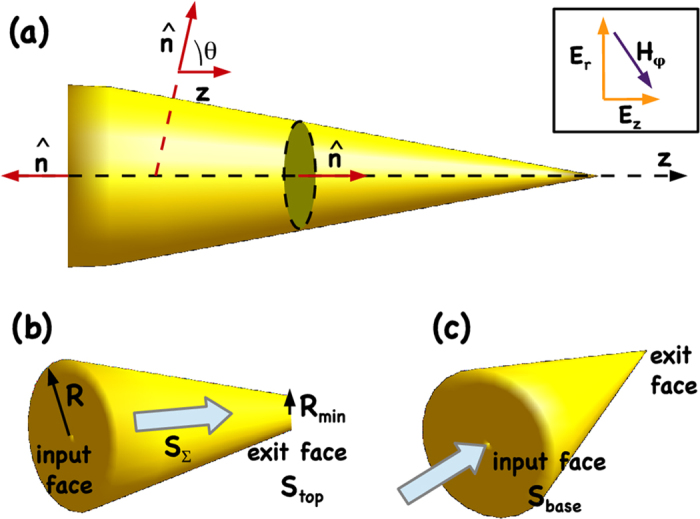
Theoretical modeling of the nanocone. (**a**) Schematic of nanocone with the normal versor 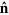 exiting from the integral surface *S*_*tot*_ (black dashed line), (**b**,**c**) Frustum and ideal nanocone, the white arrow indicates the light direction.

The theoretical calculations for the expression of the optical pressure acting on the nanocone are based on the Maxwell Stress Tensor (MST) method. The leading principle for the calculation of the optical force is the electromagnetic momentum-energy conservation. When incident light injects on a particle, the scattering and absorption processes change the light momentum that is transferred to the mechanical momentum of the particle. Integrating the change of the electromagnetic momentum on a surface containing the particle, it is possible to obtain the optical forces acting on the particle:


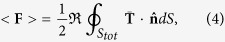


where the brackets < ... > indicate the time average over an optical cycle and 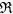 is the real part of the integration. **T** is the Maxwell Stress Tensor and gives the momentum density of the electromagnetic field in a medium with relative permittivity 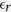 and relative permeability *μ*_*r*_,


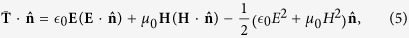



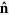
 is the normal unitary vector exiting the external surface *S*_*tot*_ = *S*_*base*_ + *S*_*top*_ + *S*_Σ_ nearly surrounding the gold nanobullet (black dashed line in Fig. 5(a)). We define *S*_*base*_, *S*_*top*_ and *S*_Σ_ as respectively the input surface, the exit surface and the lateral surface [see Fig. 5(b,c)] of the nanocone. Because the light injects along the z-direction on the input face, in Eq. (4) we specifically consider the longitudinal *z*-component of the force 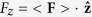. The transverse components of the forces are several order of magnitude smaller and vanish because of the cylindrical symmetry.

For a cylindrical reference system (*r*, *φ*, *z*), the SPP is a *TM*_0_ mode with components *E*_*r*_ (radial), *E*_*z*_ (along the cone axis *z*), and magnetic component *H*_*φ*_ (azimuthal)^23^,^36^, [see inset in Fig. 5(a)]. In particular, their analytical expression takes the form,


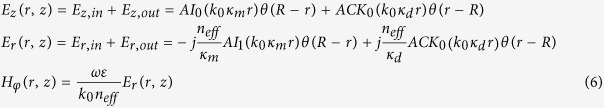


where *j* is the imaginary unit, *A* determines the amplitude of the electric field, *θ* is the Heaviside function and *C* = *I*_0_(*k*_0_*κ*_*m*_*R*)/*K*_0_(*k*_0_*κ*_*d*_*R*). We define a triad (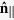, 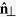, 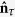) of versors along the three directions defined by *z*, *r* and *φ*, and write the electric and magnetic fields using (6),


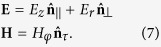


We first calculate the contribution to the opto-mechanical force deriving from the bottom (*z* = 0) *S*_*base*_ and top (*z* = *h*) *S*_*top*_ faces, then we analyze the optical force related to the lateral surface.

### Input and Exit faces

Here, we will calculate the forces acting on surfaces that are normal to the light propagation direction. With reference to Fig. 5(a), we have that *θ* = 0, 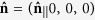 and using Eq. (7), we obtain that 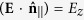, 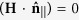. Now, we project the optical force density [Eq. (5)] along the *z*-axis obtaining:


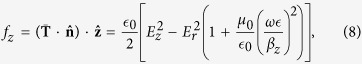


where *β*_*z*_ = *k*_0_*n*_*eff*_. We separate the inner [*r* < *r*_*c*_(*z*)] and outer [*r* > *r*_*c*_(*z*)] components of the *TM*_0_ mode in Eq. (6) and average over time, taking the real part, we obtain for a given *z*


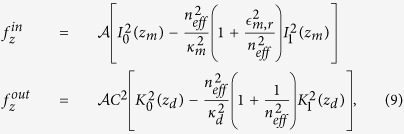


with 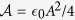, *z*_*m*_ = *k*_0_*κ*_*m*_*r* and *z*_*d*_ = *k*_0_*κ*_*d*_*r*, where 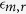 is the relative permittivity of the cone and 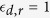. Finally, the overall force through the exit and input faces of the cone with base radius *R* and top radius *R*_*min*_ is then given by


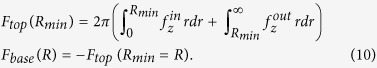


For an ideal cylinder, the two components of Eq. (10) cancel each other out. In a real system, the partial reflection gives a residual term (as seen in Fig. 3a,d).

### Lateral surface

For what concerns *S*_Σ_, we distinguish the case of the cylinder from the frustum cone. In the former case, 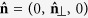 and *θ* = *π*/2. And when we project the optical force density on the *z*-axis, the result 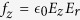 is zero. In the case of a frustum cone (or ideal cone), the lateral contribution of the force is relevant and cannot be neglected. We define 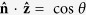 and 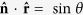 while 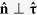 and integrate the flux of the MST through the element of surface *d*Σ = *r*_*c*_(*z*)*dφdz*/sin*θ*, where *r*_*c*_(*z*) is the local radius of the cone at fixed *z*. We obtain for the lateral density optical force


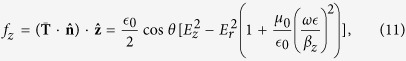


where we have used the relations 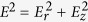 and 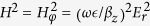. We must evaluate the integral on the external lateral surface of the cone but we observe that from the transcendental equation [see Eq.(1)], *E*_*z*,*out*_ = *E*_*z*,*in*_ while the radial component of the electric field is discontinuous 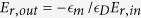. Finally, using the expressions in Eq. (6), we obtain


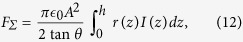


where


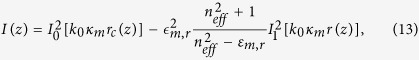


with *θ* = *π*/2 − arctan[(*R* − *R*_*min*_)/*h*].

By using Eqs. (10) and (12), as shown in Fig. 3, we obtain the overall optical force for the gold nanobullet.

## Additional Information

**How to cite this article**: Folli, V. *et al.* Laser propulsion of nanobullets by adiabatic compression of surface plasmon polaritons. *Sci. Rep.* **5**, 17652; doi: 10.1038/srep17652 (2015).
